# Genome sequencing and comparative genomic analysis of bovine mastitis-associated *Staphylococcus aureus* strains from India

**DOI:** 10.1186/s12864-022-09090-7

**Published:** 2023-01-25

**Authors:** Ramamoorthy Sivakumar, Parameswaran Sree Pranav, Madhavi Annamanedi, S. Chandrapriya, Shrikrishna Isloor, Jeyaprakash Rajendhran, Nagendra R. Hegde

**Affiliations:** 1 Department of Genetics, School of Biological Sciences, Madurai Kamaraj University, Madurai, 625021 India; 2 National Institute of Animal Biotechnology, Hyderabad, 500032 India; 3 Department of Veterinary Microbiology, Veterinary College, Karnataka Veterinary, Animal and Fisheries Sciences University, Bengaluru, 560024 India

**Keywords:** Bovine mastitis, *Staphylococcus aureus*, MLST, cgMLST, Resistome, Virulome

## Abstract

**Background:**

Bovine mastitis accounts for significant economic losses to the dairy industry worldwide. *Staphylococcus aureus* is the most common causative agent of bovine mastitis. Investigating the prevalence of virulence factors and antimicrobial resistance would provide insight into the molecular epidemiology of mastitis-associated *S. aureus* strains. The present study is focused on the whole genome sequencing and comparative genomic analysis of 41 mastitis-associated *S. aureus* strains isolated from India.

**Results:**

The results elucidate explicit knowledge of 15 diverse sequence types (STs) and five clonal complexes (CCs). The clonal complexes CC8 and CC97 were found to be the predominant genotypes comprising 21 and 10 isolates, respectively. The mean genome size was 2.7 Mbp with a 32.7% average GC content. The pan-genome of the Indian strains of mastitis-associated *S. aureus* is almost closed. The genome-wide SNP-based phylogenetic analysis differentiated 41 strains into six major clades. Sixteen different *spa* types were identified, and eight isolates were untypeable. The cgMLST analysis of all *S. aureus* genome sequences reported from India revealed that *S. aureus* strain MUF256, isolated from wound fluids of a diabetic patient, was the common ancestor. Further, we observed that all the Indian mastitis-associated *S. aureus* isolates belonging to the CC97 are mastitis-associated. We identified 17 different antimicrobial resistance (AMR) genes among these isolates, and all the isolates used in this study were susceptible to methicillin. We also identified 108 virulence-associated genes and discuss their associations with different genotypes.

**Conclusion:**

This is the first study presenting a comprehensive whole genome analysis of bovine mastitis-associated *S. aureus* isolates from India. Comparative genomic analysis revealed the genome diversity, major genotypes, antimicrobial resistome, and virulome of clinical and subclinical mastitis-associated *S. aureus* strains.

**Supplementary Information:**

The online version contains supplementary material available at 10.1186/s12864-022-09090-7.

## Introduction

Mastitis accounts for significant economic losses to dairy enterprises worldwide due to reduced milk production, culling of animals, and treatment costs [1, 2]. The primary cause of mastitis is intramammary infection (IMI). *Staphylococcus aureus* is one of the major pathogens responsible for IMI, which subsequently leads to subclinical mastitis (SCM) and clinical mastitis (CM) [3, 4]. The pathogenesis of mastitis due to *S*. *aureus* is a dynamic process, which begins with the organism colonizing the teat end and subsequently spreading into the intramammary space [5, 6]. In the mammary alveolus, *S*. *aureus* adheres to and enters mammary epithelial cells, which serve as the site for multiplication, eventually resulting in a chronic IMI [7, 8]. The molecular mechanisms underlying *S*. *aureus* IMI still need to be fully deciphered [9, 10].

Molecular subtyping of *S*. *aureus* strains will be helpful to understand its epidemiology and virulence in causing mastitis. Various strategies have been used in the past few decades to study the molecular epidemiology of mastitis-associated *S*. *aureus*, including pulsed-field gel electrophoresis (PFGE) [11], random amplification of polymorphic DNA (RAPD) analysis [12], multilocus enzyme electrophoresis (MLEE) [13], multilocus sequence typing (MLST) [14], staphylococcal protein A (*spa*) typing [15], and multiple-locus VNTR (variable number of tandem repeats) analysis (MLVA) [16]. In recent years, whole-genome sequencing, employing next-generation sequencing (NGS), has become the preferred method for understanding the phylogenetic relationship, microevolution, and inter- and intra-species differentiation [17].

Comparative genomic analysis has shown that the accessory genome plays a significant role in the genetic diversity of *S*. *aureus* clones [18]. The accessory genome of *S*. *aureus* is predominantly comprised of elements acquired through horizontal gene transfer (HGT), such as phage genes, virulence factors, and antimicrobial resistance (AMR) genes [6]. A suite of virulence determinants has been identified in *S*. *aureus* strains isolated from bovine mastitis; most genes are associated with host immune evasion during infection [6, 19]. *S*. *aureus* also carries several AMR genes in its arsenal, such as *blaZ*, *tetM*, and *mecA* [20].

Several genetic lineages of mastitis-associated *S*. *aureus* have been reported worldwide [21, 22]. The *S*. *aureus* clones that cause bovine mastitis largely fall under a set of clonal complexes (CC), such as CC151, CC97, CC133, CC479, and CC771 [23, 24]. The major virulence factors and AMR genes are localized on mobile genetic elements (MGEs). A recent report suggested that CC398, the dominant livestock-associated MRSA in Europe, rapidly adapted to cause human infections [25]. Due to the clonal expansion, *S*. *aureus* strains belonging to the same CC have specific virulence gene signatures [26, 27] and restriction-modification systems that reduce HGT between different *S*. *aureus* lineages [28, 29].

From India, Sharma et al. (2015) [30] reported the draft genome sequence of a mastitis-associated *S. aureus* strain belonging to a new sequence type ST3176. We recently reported the molecular fingerprinting of 166 bovine mastitis-associated *S*. *aureus* isolates from India using PFGE, *spa* typing, and MLST [31]. Also, we identified virulence genes such as *hlg*, *tsst*, and *pvl* among various isolates using PCR-based detection [31]. The whole-genome-based approaches are expected to resolve better the molecular epidemiology as well as the potential determinants responsible for pathogenicity of *S*. *aureus*. However, no reports on the whole genome sequences (WGS) and comparative genomics of multiple isolates of mastitis-associated *S. aureus* from India are available. This study reports the WGS of 41 selected mastitis-associated *S*. *aureus* strains isolated from India. Comparative genome analysis was performed to understand their genetic diversity, virulence factors, AMR genes, and clonal types. Also, phylogeny analysis of all *S*. *aureus* genomes reported hitherto from India was performed to discriminate genotypes of the livestock- and human-associated *S*. *aureus* strains.

## Methods

### Whole genome sequencing, assembly, and annotation

Forty-one bovine mastitis-associated *S*. *aureus* strains, which had been curated at the Department of Microbiology, Karnataka Veterinary, Animal & Fisheries Sciences University, Bengaluru, and the National Institute of Animal Biotechnology, Hyderabad [31], were used in this study. The isolates were selected to represent different host species (34 from cows, 7 from buffaloes), locations (one each from Gujarat and Uttar Pradesh states, two from Telangana state, 3 from Meghalaya state and 34 from Karnataka state), disease manifestations (23 subclinical, 4 clinical and 14 with unrecorded severity), setting (12 from urban, 14 from suburban or town and 15 from village) and time of sample collection (11 with no record, 8 from 2009, 2 from 2010, 13 from 2011, 7 from 2013) (Table S1). Genomic DNA from each strain was extracted using DNeasy blood and tissue kit (Qiagen) following the manufacturer’s instructions. The RNA-free DNA was used for library preparation and sequencing using Illumina Nextseq 500. The adaptor sequences were trimmed using the Trimomatic tool [32] and the adaptor-free high-quality reads were assembled using SPAdes v 3.11.1 [33]. The assembled genome sequences were annotated using Rapid Annotations using Subsystems Technology (RAST) server [34].

### Pan-genome analysis

All the genome sequences were re-annotated using the Prokka v1.14.6 [35], and the GFF3 files were used as input for the pan-genome analysis using Roary v3.13.0 [36] and BPGA [37].

### Multilocus sequence typing

Using the whole-genome sequences, in silico multilocus sequence typing (MLST), based on the seven housekeeping genes, *arcC*, *aroE*, *glpF*, *gmk*, *pta*, *tpi*, and *yqi*, was carried out using MLST v2.0 webserver [38]. The allelic profiles were compared with the PubMLST database and the sequence types (STs) [39]. Globally optimized eBURST analysis was performed to cluster MLSTs into clonal complexes (CC) using the Phyloviz software v2.0 [40].

### Core-genome multilocus typing

The core-genome multilocus typing (cgMLST) was performed in Bionumerics v8.0 [41] using the scheme available for *S. aureus*, which is based on 1,861 core loci. The clustering was performed in the categorical-difference coefficient method, and the minimum spanning tree (MST) was constructed using the unweighted pair group method with arithmetic mean (UPGMA) algorithm.

### *Spa* typing

The *Staphylococcus* protein A (*spa*) typing was done using the spaTyper v1.0 webserver of the Center for Genomic Epidemiology [42].

### Identification of SNPs

The whole-genome SNP-based phylogenetic tree was constructed using CSI Phylogeny v1.4, a web-based tool [43]. The genome sequences of the 41 strains were mapped against the reference genome sequence (K5) using BWA v. 0.7.2 tool of the CSI Phylogeny. The depth of each mapped position was calculated using genomeCoverageBed of the BEDTools v. 2.16.2. and the SNPs were called using the mpileup part of SAMTools v. 0.1.18. The SNPs with minimal values of 10X depth at SNP positions, 10% of relative depth at SNP positions, 10 bp distance between SNPs, SNP quality of 30, map reading quality of 25, and Z score 1.96 were considered.

The CSI Phylogeny v1.4 calls SNPs, filters the SNPs fulfilling the above parameters, does site validation, and makes a maximum likelihood phylogenetic tree based on the concatenated alignment of the SNPs using the FastTree 2. The Newick file of the SNP tree was visualized in iTOL v6 [44], and the information regarding sequence type, clonal complex, spa types, clinical/ subclinical mastitis, isolation location was added manually.

### Identification of antimicrobial resistance (AMR) genes and virulence factors

The presence of *mecA/mecC* genes in all the 41 genome sequences was screened using the SCCmecFinder v1.2 [45]. The AMR genes were identified using the resistance gene identifier (RGI) with default parameters in the comprehensive antibiotic resistance database (CARD)[46]. The virulence factor (VF) associated genes were identified using the VFanalyzer in the virulence factor database (VFDB) [47].

### Data availability

Genome sequences used in this study are available in NCBI with the following accession numbers: JAHSUU000000000, JAHSUV000000000, JAHSUK000000000, JAHSUR000000000, JAHSUQ000000000, JAHSUJ000000000, JAHRIE000000000, JAHNVJ000000000, JAHNVI000000000, JAHNVH000000000, JAHNVG000000000, JAHNVE000000000, JAHNVF000000000, JAHNVA000000000, JAHNVC000000000, JAHNVD000000000, JAHNVB000000000, JAHNUY000000000, JAHNUZ000000000, JAHNUW000000000, JAHNUX000000000, JAHNUS000000000, JAHNUT000000000, JAHNUQ000000000, JAHMIQ000000000, JAHMIP000000000, JAHMIR000000000, JAHMIO000000000, JAHLZV000000000, JAHLZT000000000, JAHLZU000000000, JAHLZO000000000, JAHLZM000000000, JAHLZK000000000, JAHLZL000000000, JAHLZJ000000000, JAHLZI000000000, JAHLZS000000000, JAHLZR000000000, JAHLZQ000000000, JAHLZP000000000.

## Results

### Genome sequencing and general features of mastitis-associated *S. aureus* strains

In this study, whole genomes of 41 bovine mastitis-associated *S*. *aureus* strains from India were sequenced using the Illumina platform.. We obtained a minimum of 100X mean sequence coverage for each genome. After the read processing and the assembly of the high-quality reads, the draft genomes were obtained from 26 to 132 contigs. The mean genome size was 2.7 Mbp, and the average total GC content was 32.7%. The summary of genome sequences is given in Table S1. The SCCmecFinder screening confirmed that all the strains were methicillin-susceptible *S*. *aureus* (MSSA).

### Pan-genome analysis

**Fig. 1:**
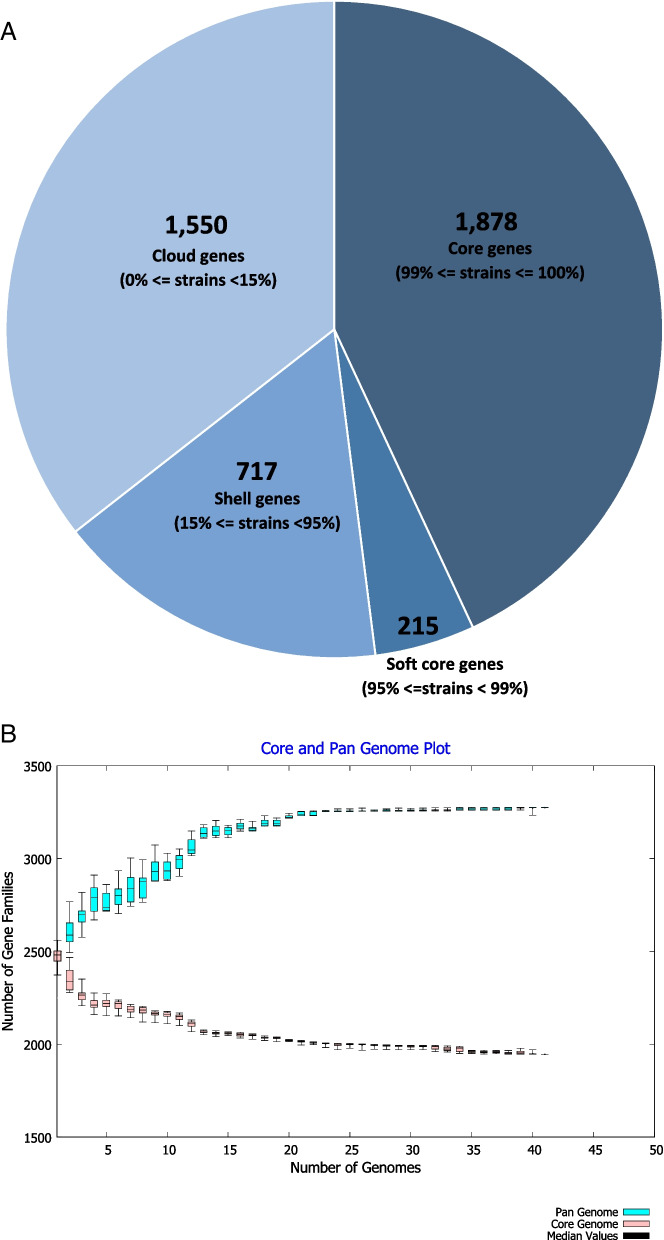
Pan-genome of 18 clinical and 23 subclinical mastitis-associated *S. aureus* isolates obtained from India. **A**- Pie chart depicting the numbers of core-, soft core-, shell- and cloud genomes. **B**- Gene family accumulation curves of the pan-genome and the core-genome

The pan-genome of 41 strains of *S. aureus* represented a total of 4,360 genes. The core genome consisted of 1,878 genes (shared by > 99% of the strains). The soft-core-, shell-, and cloud- genomes comprised 215, 717, and 1,550 genes, respectively. The power-law regression plot shows that the pan-genome of bovine mastitis-associated *S. aureus* strains from India is almost closed (Fig. 1). The power-law equation (“f(x) = a × xb”, where the f(x) denotes the number of gene families with a total expansion rate of b = 0.0817389) also indicated that the pan-genome is almost closed. This suggests that the genome sequencing effort on mastitis-associated *S. aureus* is adequate, and adding a newer genome sequence is not expected to significantly increase the pan-genome size.

### Identification of sequence types based on MLST

The in silico MLST clustered the 41 strains into 15 sequence types (STs). The majority of the isolates were assigned to the ST2454 (*n* = 17), followed by the ST2459 (*n* = 5) and ST4968 (*n* = 4). The sequence types, ST467, ST5, and ST672 were represented by two strains each, whereas ST243, ST5360, ST6, ST4976, ST2453, ST1687, ST580, and ST97 were represented by one strain each. The STs could be grouped into five different clonal complexes (CC). The goeBURST analysis clustered ST2454 and ST4968 strains into CC8, making it the largest group (21 out of the 41 strains). The sequence types ST2459, ST4967, ST1687, ST2459, and ST97, were clustered into CC97, representing the second largest group (*n* = 10). The ST5360 and ST5098 clustered into CC1. The ST5 and ST6 belonged to CC5, and ST243 belonged to CC30. The sequence types ST672, ST580, and ST4976 do not belong to any known CCs.

### Genome-wide SNP-based phylogeny analysis

**Fig. 2:**
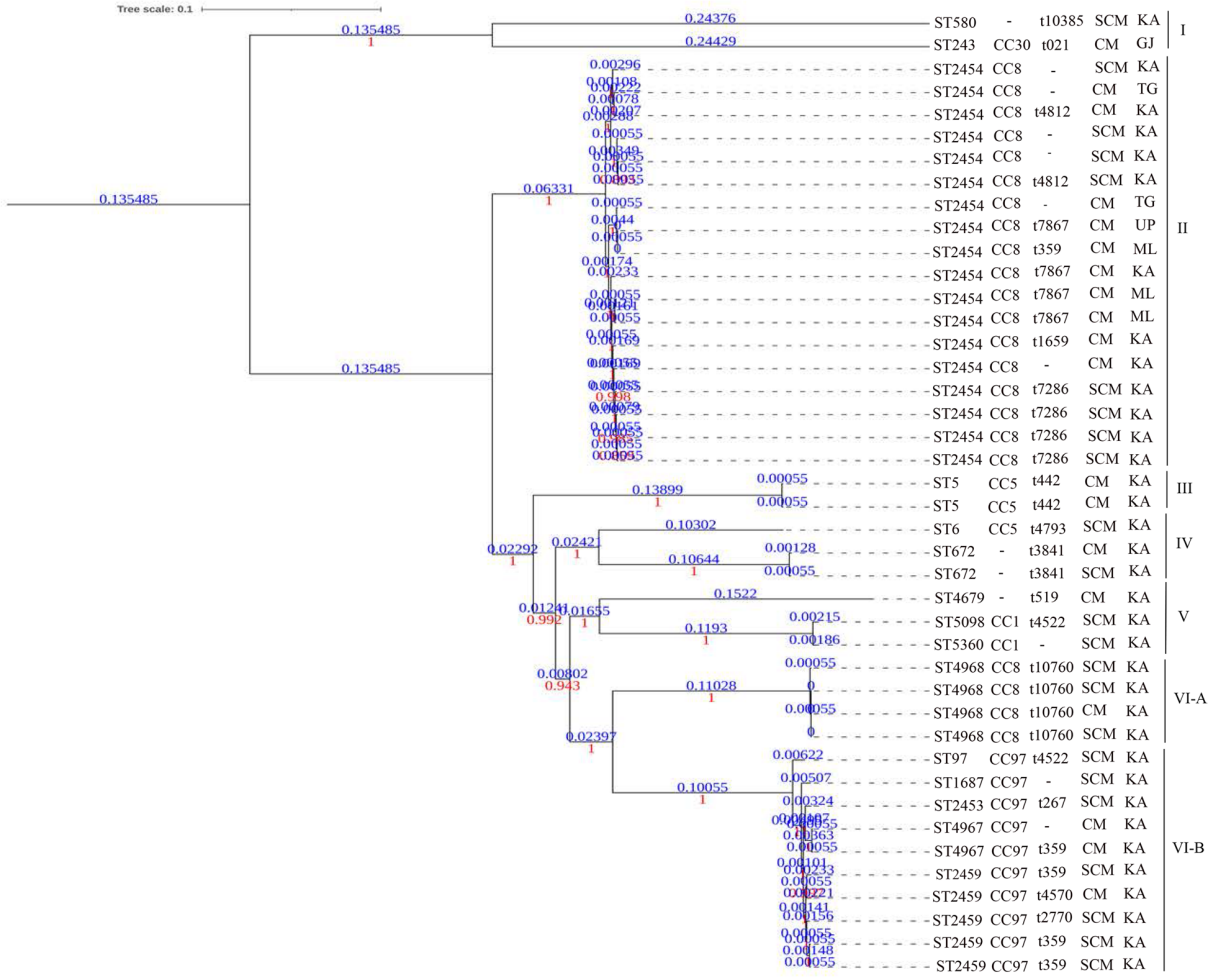
The genome-wide SNP-based maximum likelihood phylogenetic tree of mastitis-associated *S. aureus* isolates obtained from India. The *S. aureus* strain K5 (NCBI accession No: NZ_CP020656.1) was used as the reference genome to map and screen the SNPs. The phylogenetic tree was constructed using The CSI Phylogeny v1.4 and visualized using iTOL v6. The scale bar indicates 0.1 substitutions per nucleotide position. The strain names, sequence types (STs), clonal complexes (CCs), spa types, clinical (CL) or subclinical (SCL) mastitis, and five different states in India (GJ-Gujarat, KA-Karnataka, ML-Meghalaya, TG-Telangana, UP-Uttar Pradesh) from where the strains were isolated are indicated, respectively

When all the 41 strains were subjected to genome-wide SNP prediction against the reference genome using the CSI Phylogeny v1.4 tool, 2,293,099 variant positions were identified with 84.4% coverage. The genome-wide SNP-based phylogenetic tree clustered the 41 strains into six major clades (Fig. 2). The clade I, which clustered along with the reference genome (K5), was the largest clade, represented by 17 strains. All the 17 strains belonged to ST2454 and CC8. Clade II consisted of two strains belonging to ST580 and ST243. Clade III consisted of two strains belonging to ST5 and CC5. The clade IV was represented by three strains belonging to ST6 and ST672. Clade V consisted of three strains belonging to ST4679, ST5098, and ST5360. Of these, the latter two STs belong to CC1. The clade VI, the second-largest clade, was represented with 14 strains. This clade was differentiated into two sub-clades, VIA and VIB. The clade VIA was represented by four strains belonging to ST4868 and CC8. The clade VIB was represented with ten strains belonging to four different sequence types (ST97, ST2453, 4967 2459) of CC97, the second largest group in this study.

### *Spa* types

We identified sixteen different *spa* types among the 41 genomes studied. Among 17 ST2454 (CC8) genomes, five *spa* types, t7286, t1659, t7867, t359 and t4812 were identified and five were untypeable. All four ST4968 (CC8) belonged to the *spa* type t10760. Among 10 genomes belonging to CC97, two were *spa* untypeable, t359 was identified in four genomes, and t4570 and t2770 were found in one genome each. The strain K39 of CC1 was also untypeable. The ST243 (CC30) genome belonged to *spa* type t021 and two ST5 (CC5) genomes belonged to t442. The *spa* types t267, t10385, t021, t1659, t4793, t3841 and t519 were found in one genome each.

Overall, no association could be found with respect to clinical (CM) and sub-clinical (SCM) mastitis-associated strains with any of the typing tools, such as MLST sequence types (STs), clonal complexes (CCs), or the *spa* types. Similarly, no association could be found with respect to the States in India from where the strains were isolated. The genome-wide SNP-based tree clustered the strains based on the STs and the CCs, however irrespective of the *spa* types, SCM/CM and geographical location of the isolation.

### Core-genome MLST-based phylogeny analysis of *S. aureus* genome sequences reported from India

**Fig. 3:**
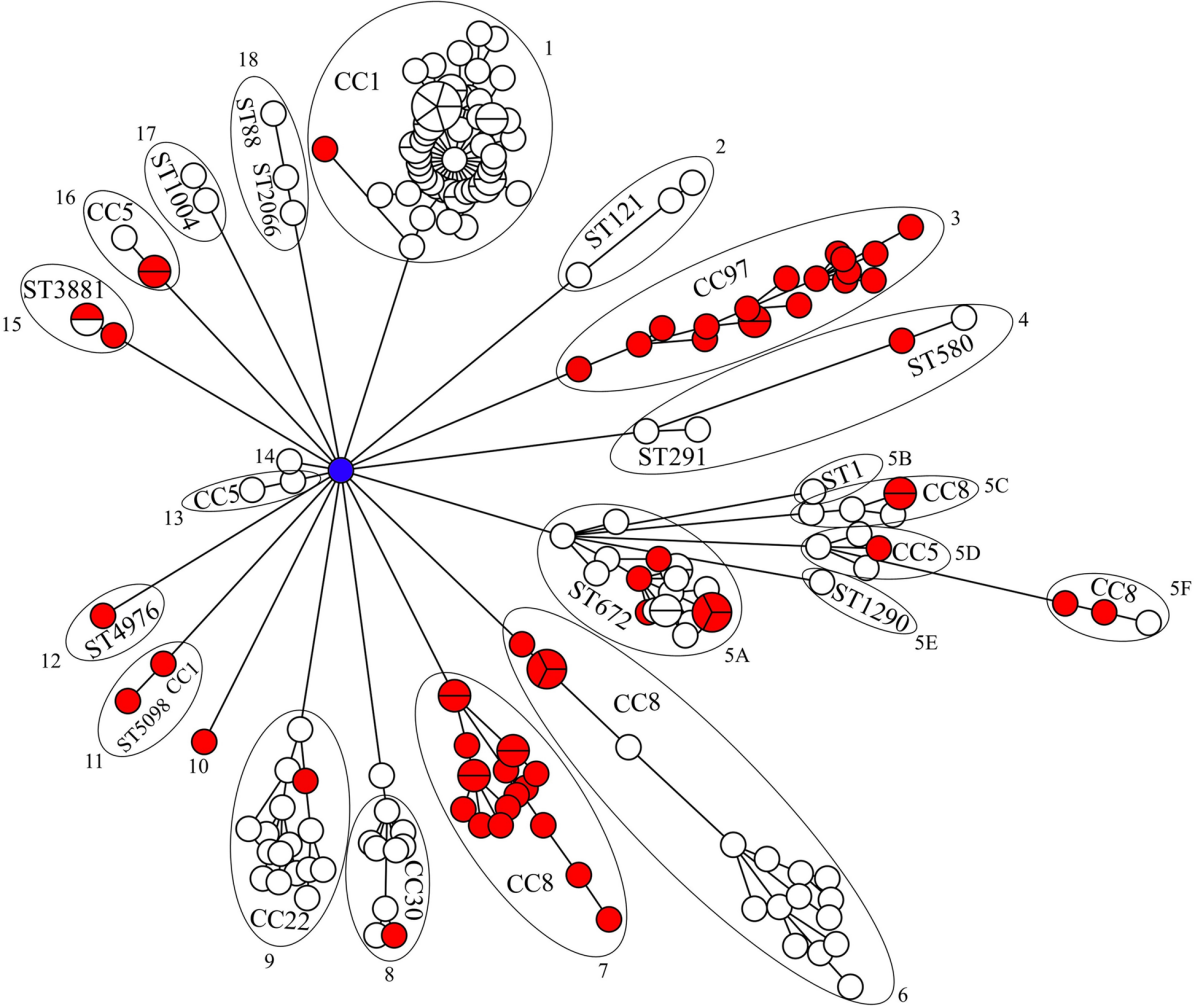
The minimum spanning tree based on the cgMLST profiles of 198 *S. aureus* genome sequences reported from India. The clade numbers, along with the names of the clonal complexes (CCs) or the sequence types (STs), if CC could not be assigned, are shown. The red nodes indicate the mastitis-associated *S. aureus* isolates. The blue node in the middle highlights the strain MUF256

We performed a core-genome MLST (cg-MLST) analysis of 198 *S. aureus* genome sequences reported hitherto from India, including the 41 genomes used in this study to understand the evolutionary relationship between bovine mastitis- and human-associated *S. aureus* genomes. Of these, 64 strains were mastitis-associated, and 122 strains were isolated from various human clinical specimens. Other strains were isolated from dogs (*n* = 2), fishes (*n* = 3), shrimp (*n* = 3), water samples (*n* = 2), and soil sediments (*n* = 2) (Supplementary Table S2). Overall, 40 STs and six CCs were assigned to these genomes (Fig. 3). The minimum spanning tree showed 18 clades. Surprisingly, all the clades were rooted in a human wound isolate, *S. aureus* MUF256. This strain was isolated in 2012 from the foot ulcer of a diabetic patient from Karnataka, India [48]. This strain belongs to the *spa* type t127. However, the ST and the CC could not be assigned to this strain. The mastitis-associated *S. aureus* genomes reported in this study and previous studies were distributed in 14 of 18 clades and clustered with human and other isolation sources. Notably, clades 3 and 7 were represented entirely by the mastitis-associated *S. aureus* strains belonging to CC97 and CC8, respectively. In addition, the CC8 strains were found in clades 5C, 5F, and 6, along with some human-associated strains.

**Fig. 4:**
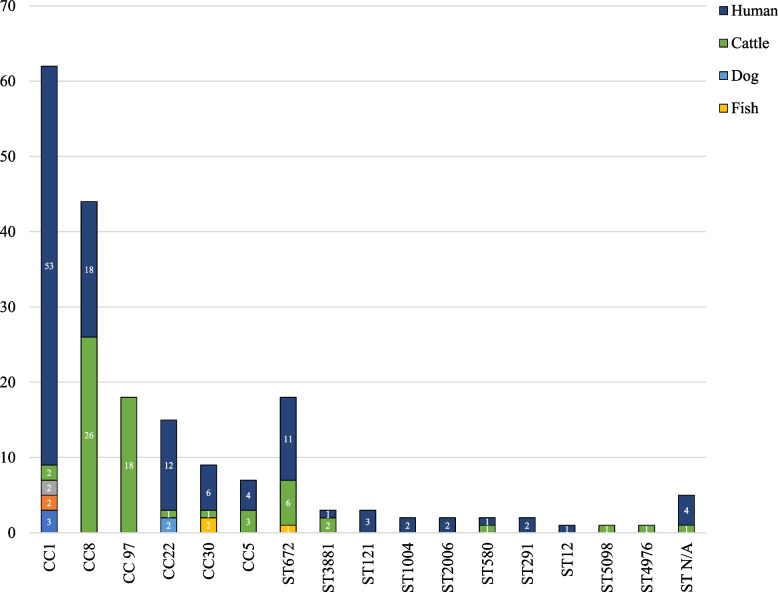
Isolation sources and distribution of clonal complexes (CCs) and sequence types (STs) among 198 *S. aureus* genome sequences reported from India

The distribution of CCs and STs among various isolation sources of 198 *S. aureus* genomes is shown in Fig. 4. CC1 was the largest clade comprising 56 genomes. Among them, 48 were of human origin, three from shrimp, two from water samples, two from soil sediments, and only one was a mastitis-associated *S. aureus*. The second-largest group was the CC8 comprising 44 genomes. Of these, 26 are mastitis-associated, and 18 are human-associated. The third group contains 18 genomes, represented entirely by mastitis-associated *S. aureus*. Of these, ten genomes are from this study, and eight were previously reported. Thus, all *S. aureus* strains of CC97 with whole-genome sequences from India are mastitis-associated.

### Distribution of AMR genes among mastitis-associated *S. aureus* strains

**Fig. 5:**
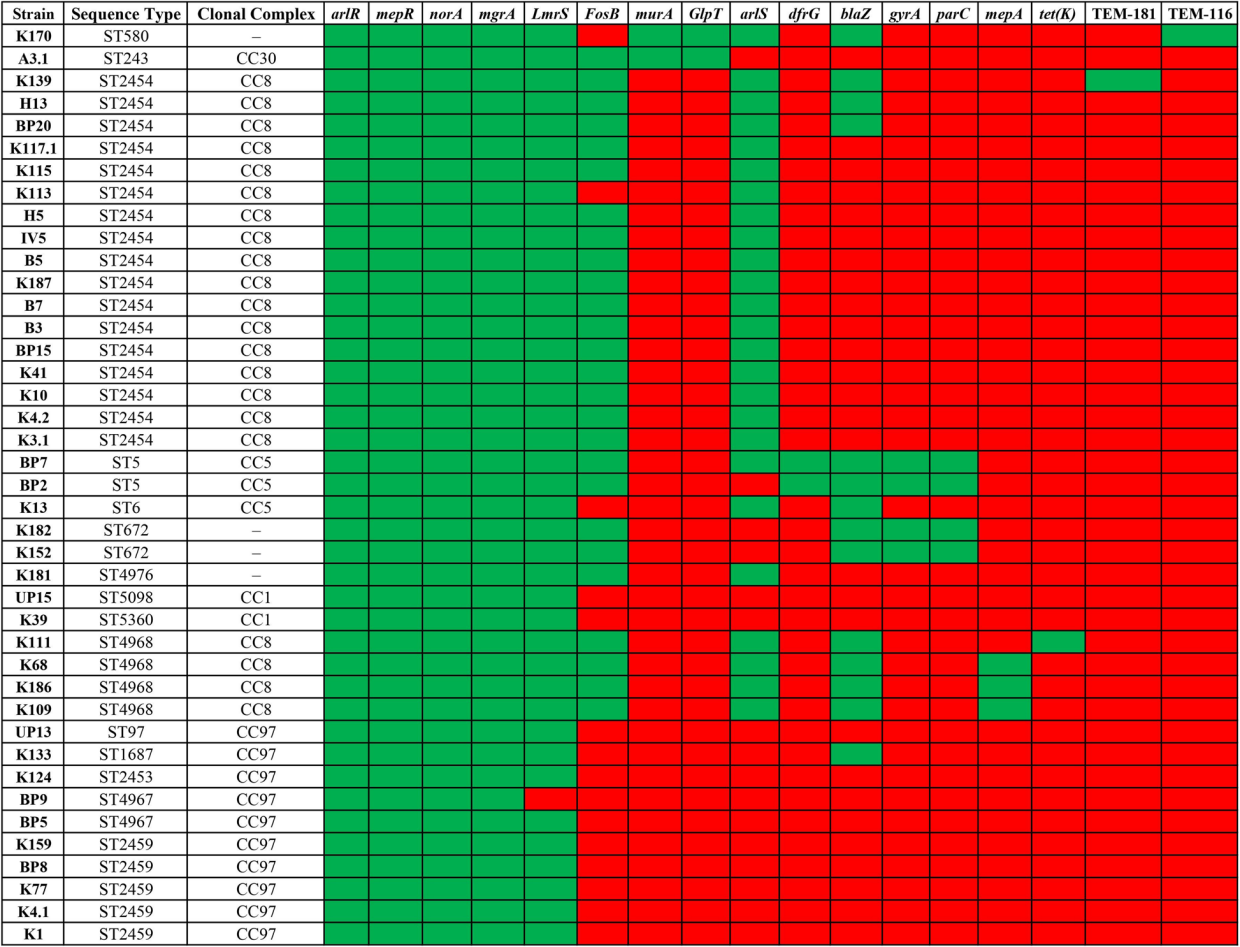
The resistome of 41 mastitis-associated *S. aureus* isolates hierarchically clustered based on the presence (green) or absence (red) of 17 antimicrobial resistance (AMR) genes

The resistance gene identifier (RGI) analysis revealed the presence of 17 different AMR genes among 41 genomes (Fig. 5). Of these, four AMR genes, *arlR* (response regulator ArlR), *mepR* (multidrug efflux transporter transcriptional repressor MepR), *norA* (quinolone resistance protein NorA), *mgrA* (HTH-type transcriptional regulator MgrA), were found in all genomes. The *lmrS* (major facilitator superfamily multidrug efflux pump) was present in all the genomes except the strain BP9. The *fosB* (metallothiol transferase FosB) was present in all 21 strains belonging to CC8 (ST2454 = 17; ST4968 = 4), two strains belonging to CC5 (ST5), two strains belonging to ST672, and a strain belonging to CC30 (ST243). Both *murA* (UDP-N-acetylglucosamine 1-carboxyvinyltransferase) and *GlpT* (glycerol-3-phosphate transporter) genes were found only in two strains belonging to CC30 and ST580, respectively. The *arlS* (response sensor ArlS) was found in 25 genomes, although its response regulator was found in all 41 genomes. The *dfrG* (dihydrofolate reductase) was found in all three strains belonging to CC5 (ST5 = 2; ST6 = 1). The *blaZ* (β-lactamase) was found in 15 strains; six belong to CC8, and three belong to CC5 (ST5 = 2; ST6 = 1). Both *gyrA* (DNA gyrase subunit A) and *parC* (DNA topoisomerase four subunit A) were present only in strains belonging to CC5 (ST5 = 2; ST6 = 1) and ST672. The *mepA* (penicillin-insensitive murein endopeptidase) was found in three out of four ST4968 (CC8) strains. Furthermore, *tet*(*K*) (tetracycline resistance protein), TEM-116, and TEM181 (TEM β-lactamases) were present in only one strain, K111 (ST4968), K170 (ST580), and K139 (ST2454), respectively.

### Distribution of virulence factors among mastitis-associated *S. aureus* strains

**Fig. 6:**
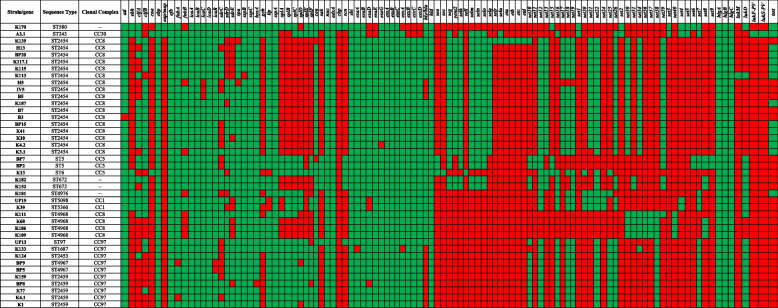
The virulome of 41 mastitis-associated *S. aureus* isolates hierarchically clustered based on the presence (green) or absence (red) of 108 virulence genes

A total of 108 virulence factors were detected among 41 mastitis-associated *S. aureus* genomes. These virulence factors could be categorized into five functional categories, as shown in Fig. 6.

- 1. Adherence-related genes
  The genes coding for elastin binding protein (*ebp*), fibrinogen binding protein (*efb*), and intercellular adhesins (*icaA*, *icaB*, and *icaD*) were identified in all 41 genomes. The gene coding for autolysin (*atl*) was found in all genomes except B3. The cell wall-associated fibronectin-binding protein-coding gene (*ebh*) was found in genomes belonging to CC1, CC30, ST672, ST580, ST4976, and ST6. The clumping factor A gene (*clfA*) was found in CC30, ST580, ST5, CC1, ST2453, ST4976, 11 genomes of CC8, and one genome of ST672. Similarly, the *clfB* gene was identified in genomes belonging to CC1, ST5, ST4976, and 16 genomes of ST2454 (CC8), one genome of ST4968 (CC8), and three genomes of CC97. The fibronectin-binding protein gene (*fnbA*) was found in all genomes except two CC97 genomes. Another fibronectin-binding protein-coding gene (*fnbB*) was absent in all four genomes of ST4968 (CC8), two genomes of ST2454 (CC8), and one genome each belonging to ST580 and ST4976. Among the intercellular adhesion coding genes, the *icaC* gene was present in all genomes except three strains of ST2454 (CC8). The intercellular adhesin regulator gene (*icaR*) was found in all genomes except two strains of ST4967 (CC97). The genes encoding Ser-Asp rich fibrinogen-binding proteins (*sdrC*, *sdrD* and *sdrE*) were present in 24 to 31 different genomes. The extracellular adherence protein/MHC analogous protein (*eap/map*) was detected only in two genomes belonging to ST240 and ST580. Notably, these two genomes formed clade II in the genome-wide SNP-based tree (Fig. 2). The staphylococcal protein A (*spa*) was not found in two genomes.
- 2. Genes encoding enzymes
  Fifteen different virulence-associated enzymes were predicted among 41 genomes. The cysteine protease (*sspC*), serine V8 protease (sspA), and thermonuclease (*nuc*) genes were found in all genomes. The cysteine protease (*sspB*) was found in all genomes except two strains, i.e., K113 (ST2454, CC8) and K181 (ST4976, CC97). The hyaluronate lyase gene (hysA) was found in all genomes except BP8 (ST2459, CC97). The lipase gene (*geh*) was found only in 12 genomes, whereas another lipase gene (*lip*) was found in 38 genomes. All six serine protease coding genes, *splA, splB, splC, splD*, *splE*, and *splF, *were missing in genomes belonging to the CC8, the largest CC observed in this study. Staphylocoagulase (*coa*) was found in all genomes except strains K139 (CC8) and BP7 (CC5), whereas staphylokinase (*sak*) was found only in seven genomes belonging to CC5, ST580, ST672, and ST97.
- 3. Genes encoding immune evasion proteins
  The genes responsible for immune evasion, adenosine synthase A (*adsA*)*, *and staphylococcal binder of immunoglobulin (*sbi*) were present in all genomes. The chemotaxis inhibitory protein of the *S*. *aureus* (CHIPS) coding gene was found only in the genome of K170, which belongs to ST580. The staphylococcal complement inhibitor (*scn*) gene was found in seven genomes belonging to CC5, ST580, ST672, and ST97 strains.
- 4. Genes encoding secretion systems
  The entire suite of 12 genes coding for the type VII secretion system (T7SS) was found in almost all genomes. Among them, *esaA*, *esaB*, *essA*, *essB, *and *essC* were present in all genomes. Other genes of T7SS, such as *esxA esxB*, *esxC*, *esxD,* *esaD esaE esaG* were missing in a few genomes in different permutations.
- 5. Genes encoding toxins
  The genes coding for delta hemolysin (*hld*) and gamma hemolysins (*hlgA*, *hlgB*, *and hlgC*) were found in all the 41 genomes. Similarly, the alpha-hemolysin (*hly/hla*) gene was present in all genomes except three strains of CC8.
  The genes coding for enterotoxin A (*sea*) was found only in three genomes of CC5. Similarly, the enterotoxin C (*sec*) gene was found in only one genome belonging to ST6 (CC5). The genes coding for enterotoxin G (*seg*), enterotoxin-like K (*selk*), enterotoxin-like M (*selm*), enterotoxin-like N (*seln*), enterotoxin-like O (*selo*) were found only among the strains belonging to ST2454 (CC8), ST5 (CC5) and ST243 (CC30). The enterotoxin Yent2 (*yent2*) and enterotoxin-like Q (*selq*) genes were present only among the strains belonging to ST2454 (CC8). The enterotoxin-like L (*sell*) gene was present only in strain K13 of ST6. The enterotoxin-like U (*selu*) gene was identified only in strain A3.1 of CC30. The genes coding for exfoliative toxin type A, B, C, and D (*eta*, *etb*, *etc*, and *etd*) were found only in strain K139 of ST2454.
  A total of 32 different staphylococcal exotoxin coding genes named from *set1 to set40* (Fig. 6) were identified among the 41 genomes of *S. aureus*. Each *set* gene was found in the range of 2 to 35 genomes and distributed in different combinations. Notably, multiple *set* genes were present among CC8 and CC97 strains. The gene coding for leukocidin M was found in only one strain, K113 (ST2454, CC8). However, the leukotoxin D gene was found in 21 genomes, including all strains of CC97, CC5, CC1, ST580, ST672, ST4976, and in one strain each of ST2454 (CC8) and ST4968 (CC8). The genes coding for the Panton-Valentine leucocidin, *lukF-PV*, and *lukS-PV* were found in only one strain, *S. aureus* A3.1 belonging to ST243 (CC30). The toxic shock syndrome toxin (*tsst*) was found in 14 genomes, of which 11 from ST2454, one each from ST243, ST580, and ST4679.

## Discussion

Mastitis poses a significant threat to the dairy industry and is responsible for enormous revenue losses worldwide. *Staphylococcus aureus* is associated with a large proportion of subclinical and clinical mastitis cases. The pathogenesis of *S*. *aureus* is a dynamic process that relies on numerous factors such as host genetic composition and immune response, geographical influences, virulence factors (VFs), and genetic variability of the bacterium. Understanding these factors through a comprehensive genome analysis of mastitis-associated* S*. *aureus* strains from different geographical regions could help to assess the risk of infection, disease manifestation, and transmission dynamics and improve the therapeutic interventions in cattle. In this direction, we sequenced the whole genome of 41 mastitis-associated *S. aureus* strains isolated from India and identified the STs and CCs. Also, we have determined the VFs and AMR genes distributed among the genomes.

All the strains used in this study were methicillin-sensitive *S*. *aureus* (MSSA), lacking the *mecA* and *mecC* genes in their genomes. Earlier, Li et al. (2017) [49] reported that 93.4% of isolates associated with bovine mastitis in eastern regions of China were the MSSA. The MLST grouped our 41 mastitis-associated *S*. *aureus* strains into 15 STs. The majority of the strains belonged to ST2454 (41%), followed by ST2459 (12%), which is in agreement with our earlier report [31]. The 15 STs could be further clustered into five CCs (CC8, CC97, CC1, CC5, and CC30), among which CC8 (51%) was the predominant clonal complex, followed by CC97 (24%).

The relatedness of isolates with respect to their STs, CCs, *and spa* types are represented in Fig. 2. The SCM isolates obtained from the State of Karnataka, India, such as K41, K10, K4.2, and K3.1 belong to the same ST (ST2454), CC (CC8) and the *spa* type (t7286). Similarly, strains K111, K68, K186, and K109 from Karnataka belong to the same ST (ST4968), CC (CC8), and *spa* type (t10760). The CM strains IV5, K187, B7, and B3 belong to the same ST (ST2454), CC (CC8), and *spa* type (t7867), but these isolates were obtained from different states across India (Uttar Pradesh, Karnataka and Meghalaya). In this study, the *spa* types t7867, t7286, and t10760 were largely present. All strains with the *spa* type t7867 caused CM. Other types were found to be associated with both CM and SCM.

Previous reports suggested that CC8 predominates the highly contagious *S*. *aureus* subtype belonging to genotype B (GTB) [50, 51]. Although CC8 is considered a human clone, a close genetic relationship between mastitis strains and human isolates has been identified [52]. The cg-MLST analysis of 198 *S*. *aureus* isolates obtained from various hosts and niches reported from India delineated the CC8 strains into two distinct clusters. Cluster I consisted of a mix of strains of human and bovine origin, while cluster II comprised strains exclusively from the bovine origin (Fig. 3). Prevalence of CC8 *S. aureus* strains associated with bovine mastitis have been identified from several European countries, such as Italy, Germany, Switzerland, and Austria [21, 53, 54]. Previous reports have also suggested the possible host shift of the CC8 strains from human to bovine [52, 55]. Thus, CC8 strains reported in this study might have originated from a human clone and subsequently transferred to cattle herds causing mastitis.

Previously, we have reported that 24% of the mastitis-associated *S*. *aureus* isolates from India belonged to CC97 [31]. This notion was further buttressed by the phylogeny of all *S*. *aureus* genomes reported from India, where CC97 formed a distinct cluster of exclusively mastitis-associated strains (Fig. 3). Substantial studies provide evidence for the involvement of CC97 as one of the major CCs in bovine mastitis; it is widespread across multiple geographic locations, including the United States, Europe, South Africa, Brazil, and Japan [21, 49, 50, 56]. In addition to bovine mastitis, CC97 has been isolated from humans and other hosts [50].

The spread of AMR among pathogenic bacteria poses a significant threat to managing bovine mastitis as well as human infections caused by *S. aureus*. Identifying AMR genes is critical to determine the pathogenic potential of *S*. *aureus* during mastitis. Scanning genome sequences facilitates the identification of genetic factors implicated in antibiotic resistance [57, 58]. We identified 17 different AMR genes among 41 genomes. Genes coding for multidrug resistance (MDR) efflux pumps, such as *norA*, *mepR*, *arlR*, *mgrA*, and *lmrS* were found in all the genomes. However, their role in conferring resistance and the physiopathological consequences need further investigation. This study identified only a few AMR genes in *S. aureus* MSSA strains compared to non-aureus staphylococci (NAS), causing bovine mastitis reported earlier [59]. This finding was in accordance with previous reports, where the prevalence of AMR genes was lesser in *S*. *aureus* than in NAS strains [22, 60, 61]. However, extended-spectrum β-lactamase (ESBL) genes such as TEM116 and TEM181 were identified in one strain each. Dissemination of such strains among cattle and humans may be a potential public health threat.

Biofilm formation is a major factor in conferring or enhancing antibiotic resistance traits to bacterial pathogens; biofilm can also contribute to persistent or chronic infection [62]. The presence of *ica* operon was observed in all the *S*. *aureus* genomes (> 98%) studied here, which play a crucial role during biofilm formation in *S*. *aureus* [63, 64]. The *ica* operon also contributes to the evasion of immunological defenses, such as resistance to antimicrobial peptides and escape from phagocytosis [65]. However, the concordance between the mere presence of *ica* genes and biofilm formation is unclear. We have observed equal distribution of *icaA* and *icaD* genes among isolates able or unable to form biofilm [66]. Irrespective of the biofilm-forming ability, more than 90% of the *S. aureus* strains contain *ica* genes (unpublished data). Nevertheless, in addition to biofilm-related genes, adenosine synthase A (*AdsA*), staphylococcal protein A (*SpA*), and staphylococcal binder of immunoglobulin (*Sbi*) genes were present in several strains. These genes play significant roles in evasion from the protective immune responses of the host [67]. Together, biofilm formation and immune evasion could be potent virulence determinants in the colonization, invasion, and survival of *S. aureus* in the udder, but a systematic analysis of phenotypic virulence characteristics is needed.

The distribution of 108 virulence factors was determined across 41 genomes of *S*. *aureus* strains. Attachment and colonization on teats are the initial steps in bovine mastitis. The *S. aureus* adhesins, such as those binding to fibrinogen, fibronectin, elastin collagen, and clumping factors, play critical roles in adhesion to the host cells, followed by colonization and invasion [68]. We found that the genes coding for elastin binding protein (*ebp*), fibrinogen binding proteins (*efb, sdrC*, *sdrD* *sdrE*), fibronectin-binding protein (*ebh, fnbA, and fnbB*), intercellular adhesins (*icaA*, *icaB*, and *icaD*), clumping factors (*clfA and clfB*), were widespread among the 41 genomes. The presence of these genes authenticates the potential pathogenicity of the strains, although further in vitro and in vivo studies would be required to confirm the pathogenic phenotype.

The pathogenic potential of *S. aureus* also depends on the secretion of toxins such as hemolysins, leukotoxins, enterotoxins, and enzymes such as serine proteases, cysteine proteases, and lipases, which act as effectors during pathogenicity [69]. Multiple hemolysin genes and some enzyme-coding genes were present in almost all the 41 genomes. Genes coding for serine proteases, cysteine proteases, and lipases were found in several genomes. Similarly, the gene coding for staphylocoagulase, which can induce blood clotting by direct activation of prothrombin, was found in 39 genomes in this study. Genes coding for enterotoxins, enterotoxin-like proteins, and exfoliative toxins were found only in a few selected genomes. Also, leukocidin M and the Panton-Valentine leucocidin genes were found in only one genome each, respectively. Similarly, genes coding for adhesins and hemolysins were more prevalent, while the occurrence of enterotoxins, leukocidins, and toxic shock syndrome toxin genes was less frequently found among bovine mastitis*-*associated *S. aureus* strains isolated from Brazil [70]. In addition, genes for the type VII secretion system (T7SS) were found in several *S. aureus* strains. The T7SS exports several virulent proteins [71], plays a significant role in resistance to the host-derived antimicrobial fatty acids [72] and facilitates the secretion of nuclease and membrane-depolarizing toxins against competitor bacteria [73]. Altogether, the presence of multiple virulence factors supports the pathogenic potential of the isolates.

## Conclusion

In summary, whole genome analysis of 41 bovine mastitis-associated *S. aureus* strains isolated from India clustered these strains into 15 STs, five CCs, and 16 *spa* types. The clonal complexes CC8 and CC97 were the major group among the isolates. Notably, CC97 was confined to the bovine origin, among all *S*. *aureus* genome sequences reported hitherto from India. The antibiotic resistance genes were dispersed among the isolates irrespective of the STs and CCs. However, certain virulence factors were confined to specific STs and CCs.

## Supplementary Information

**Additional file 1:** **Table S1.** Salient features of *Staphylococcus aureus* genomes sequenced in this study

**Additional file 2.**

## Data Availability

Genome sequences used in this study are available in NCBI with the following accession numbers: JAHSUU000000000, JAHSUV000000000, JAHSUK000000000, JAHSUR000000000, JAHSUQ000000000, JAHSUJ000000000, JAHRIE000000000, JAHNVJ000000000, JAHNVI000000000, JAHNVH000000000, JAHNVG000000000, JAHNVE000000000, JAHNVF000000000, JAHNVA000000000, JAHNVC000000000, JAHNVD000000000, JAHNVB000000000, JAHNUY000000000, JAHNUZ000000000, JAHNUW000000000, JAHNUX000000000, JAHNUS000000000, JAHNUT000000000, JAHNUQ000000000, JAHMIQ000000000, JAHMIP000000000, JAHMIR000000000, JAHMIO000000000, JAHLZV000000000, JAHLZT000000000, JAHLZU000000000, JAHLZO000000000, JAHLZM000000000, JAHLZK000000000, JAHLZL000000000, JAHLZJ000000000, JAHLZI000000000, JAHLZS000000000, JAHLZR000000000, JAHLZQ000000000, JAHLZP000000000.
